# Sleep Health Disparities in Depression: The Role of Sexual Orientation, Education, and Ethnicit

**DOI:** 10.1192/j.eurpsy.2025.2311

**Published:** 2025-08-26

**Authors:** J. Kim, M. He, S. Gunturu, O. El Shahawy

**Affiliations:** 1 BronxCare Health System, Bronx; 2 New York University Grossman School of Medicine, New York, United States

## Abstract

**Introduction:**

This study explores sleep health disparities among adults with depressive episodes, focusing specifically on sexual and gender minorities (SGM). Given the high prevalence of sleep disturbances in this population, we aim to understand the influence of sexual orientation, alongside sociodemographic factors, on sleep health.

**Objectives:**

- To determine the prevalence of sleep difficulties among sexual minorities with depressive episodes.
- To assess how sociodemographic factors, including education and ethnicity, relate to sleep health in this population.

**Methods:**

Using data from the 2020-2021 National Survey on Drug Use and Health (NSDUH), we analyzed a sample of 15,244 individuals who experienced depressive episodes. The study employed weighted estimates to accommodate the survey’s multistage sampling design. Descriptive statistics were used to assess the prevalence of various factors, including tobacco and nicotine use, age, gender, ethnicity, income, marital status, and education. To evaluate the relationships between these factors and sleep difficulties, we utilized generalized linear models with Poisson distribution and log-link function to estimate adjusted prevalence ratios for each covariate.

**Results:**

The study identified notable disparities in sleep health among individuals with depressive episodes based on sexual orientation and sociodemographic factors. Gay/lesbian individuals and bisexual individuals were both found to have a higher likelihood of reporting sleep difficulties compared to heterosexuals, with an increased prevalence of 1.06 times (p = 0.038 for gay/lesbian and p = 0.009 for bisexual). Educational attainment appeared to play a significant protective role; those with a college degree or higher were 0.89 times less likely to report sleep difficulties than individuals without a high school diploma (p < 0.001). Additionally, ethnicity influenced sleep health, with Hispanic individuals being 1.05 times more likely to report sleep issues than non-Hispanic Whites (p = 0.015).

**Image 1:**

**Figure:**
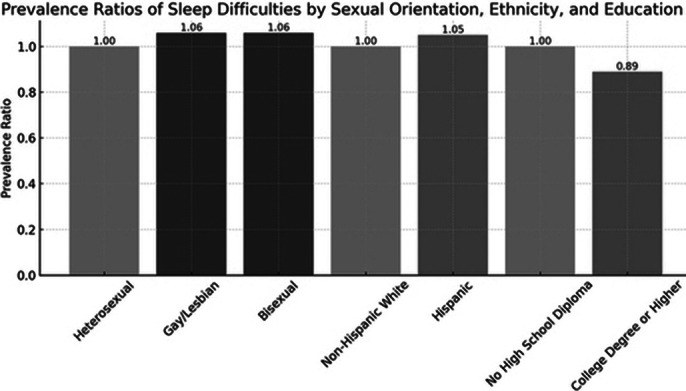

**Conclusions:**

The findings emphasize the presence of sleep health disparities among sexual and gender minorities experiencing depressive episodes. Gay/lesbian and bisexual individuals face a higher risk of sleep difficulties, highlighting the need for mental health interventions that are sensitive to sexual orientation. The protective effect of higher educational attainment suggests that enhancing access to education and related resources may improve sleep health outcomes. The increased prevalence of sleep difficulties among Hispanic individuals points to the need for culturally tailored approaches in mental health care. Addressing these disparities through individualized and culturally sensitive therapeutic strategies can contribute to better sleep health and overall well-being for these populations, underlining the importance of integrated, comprehensive care in managing depressive disorders.

**Disclosure of Interest:**

None Declared

